# Thyroid Cancer: The Quest for Genetic Susceptibility Involving DNA Repair Genes

**DOI:** 10.3390/genes10080586

**Published:** 2019-08-01

**Authors:** Luís S. Santos, Bruno Costa Gomes, Hélder N. Bastos, Octávia M. Gil, Ana Paula Azevedo, Teresa C. Ferreira, Edward Limbert, Susana N. Silva, José Rueff

**Affiliations:** 1 Centre for Toxicogenomics and Human Health, Genetics, Oncology and Human Toxicology, NOVA Medical School|Faculdade de Ciências Médicas, Universidade Nova de Lisboa, 1169-056 Lisboa, Portugal; 2 Universidade Católica Portuguesa, Center for Interdisciplinary Research in Health (CIIS), Institute of Health Sciences (ICS), 3504-505 Viseu, Portugal; 3 Department of Pneumology, Centro Hospitalar São João, 4200–319 Porto, Portugal; 4 Faculdade de Medicina, Universidade do Porto, 4200-319 Porto, Portugal; 5 IBMC/i3S - Instituto de Biologia Molecular e Celular/Instituto de Investigação e Inovação em Saúde, Universidade do Porto, 4200-135 Porto, Portugal; 6 Centro de Ciências e Tecnologias Nucleares, Instituto Superior Técnico, Universidade de Lisboa, 2695-066 Bobadela LRS, Loures, Portugal; 7 Department of Clinical Pathology, Hospital São Francisco Xavier, 1449-005 Lisboa, Portugal; 8 Serviço de Medicina Nuclear, Instituto Português de Oncologia de Lisboa (IPOLFG), 1099-023 Lisboa, Portugal; 9 Serviço de Endocrinologia, Instituto Português de Oncologia de Lisboa (IPOLFG), 1099-023 Lisboa, Portugal

**Keywords:** Thyroid cancer, DNA repair, genetic susceptibility, genetic markers, SNPs

## Abstract

The incidence of thyroid cancer (TC), particularly well-differentiated forms (DTC), has been rising and remains the highest among endocrine malignancies. Although ionizing radiation (IR) is well established on DTC aetiology, other environmental and genetic factors may also be involved. DNA repair single nucleotide polymorphisms (SNPs) could be among the former, helping in explaining the high incidence. To further clarify the role of DNA repair SNPs in DTC susceptibility, we analyzed 36 SNPs in 27 DNA repair genes in a population of 106 DTCs and corresponding controls with the aim of interpreting joint data from previously studied isolated SNPs in DNA repair genes. Significant associations with DTC susceptibility were observed for *XRCC3* rs861539, *XPC* rs2228001, *CCNH* rs2230641, *MSH6* rs1042821 and *ERCC5* rs2227869 and for a haplotype block on chromosome 5q. From 595 SNP-SNP combinations tested and 114 showing relevance, 15 significant SNP combinations (*p* < 0.01) were detected on paired SNP analysis, most of which involving *CCNH* rs2230641 and mismatch repair variants. Overall, a gene-dosage effect between the number of risk genotypes and DTC predisposition was observed. In spite of the volume of data presented, new studies are sought to provide an interpretability of the role of SNPs in DNA repair genes and their combinations in DTC susceptibility.

## 1. Introduction

Thyroid cancer (TC) is the most common endocrine malignancy and its increasing incidence raises concern. It is two to four times more frequent in women than in men and one of the most common malignancies in adolescent and young adults, ages 15–39 years, the median age at diagnosis being lower than that for most other types of cancer [1,2]. Papillary (PTC) and follicular (FTC) thyroid cancer, representing 85–90% and 5–10% of cases, respectively, are the most common histological varieties and are often collectively referred to as well-differentiated thyroid carcinoma (DTC). In contrast to anaplastic thyroid cancer (ATC), DTC prognosis is generally good, with high long-term survival and low disease-specific mortality [3,4]. 

DTC aetiology is multifactorial, resulting from the interplay between genetic and environmental factors: exposure to ionizing radiation (IR), particularly during childhood, remains the best-established modifiable risk factor, despite others – such as dietary habits (e.g., iodine intake), obesity and xenobiotic exposure – have also been proposed [2,4,5]. The importance of hereditary factors on DTC susceptibility is evidenced from familial studies demonstrating high disease risk among first-degree relatives and placing DTC as one of the cancers with higher heritability [6]. So far, the most robust evidence – provided by several genome wide association studies (GWASs), with independent replication across different populations – establishes markers at 9q22.33 (*FOXE1*), 14q13.3 (*NKX2-1*), 2q35 (*DIRC3*), 8p12 (*NRG1*) and 1q42.2 (*PCNXL2*) as the strongest genetic susceptibility markers for DTC (reviewed in [6,7]). Further candidate markers such as single nucleotide polymorphisms (SNPs) within genes involved in cell cycle control and apoptosis, DNA repair, intracellular signalling and transcriptional regulation have been proposed (reviewed in [8,9,10]) but many of these findings have not been properly replicated. Overall, currently proposed DTC risk markers are still largely insufficient to explain the high heritability of DTC [6]. It is possible that other, yet unidentified, genetic variants have a relevant impact on DTC susceptibility and thus explain part of the missing heritability of the disease. Their identification is therefore highly desirable.

DNA repair safeguards genomic integrity upon exposure to genotoxic agents, its absence or impairment leading to cancer-driving mutations in oncogenes or tumour suppressor genes (reviewed in [11,12]). A great number of DNA repair SNPs has been associated with cancer susceptibility (reviewed in [12,13]), strongly suggesting that such variants may, if functionally significant, modulate the individual sensitivity to genotoxic agents and, hence, contribute to cancer predisposition.

Considering the important role that IR and, possibly, other DNA damaging agents play in DTC aetiology, DNA repair SNPs could, through interference with DNA repair capacity, contribute to DTC susceptibility. Indeed, prior studies by our team do suggest that SNPs across different DNA repair pathways – e.g., *RAD51* and *XRCC3* (HR pathway), *CCNH* (NER pathway) and *MSH6* (MMR pathway) – may be implicated in TC (or, more specifically, DTC) predisposition [14,15,16,17,18]. Such studies add on to prior and subsequent work by other teams [8,12,19,20,21,22,23,24,25] that propose additional markers and reinforce the notion that DNA repair SNPs may contribute to DTC risk. However, besides being scarce, these studies provide only limited information on the impact of the studied SNP in specific subpopulations, e.g., male versus female patients or early-onset versus late-onset DTC. Considering the specificities of DTC regarding gender distribution and median age at diagnosis [1,2] such detailed analysis could prove useful. Although gene-gene interactions could be of utmost importance in the real context, possibly decisive, they have only seldom evaluated and, when considered [19,20,22,24,26,27], analyses were usually limited to the combined effect of SNPs in the same gene or in genes of the same pathway. DNA repair proteins functionally interact with each other, both within the same DNA repair pathway and across different pathways, establishing ground for additive or even multiplicative effects of different SNPs (irrespective of their pathway) on DNA repair activity and, hence, cancer risk. This has been previously demonstrated for other types of cancer such as breast cancer [28,29,30] and, most likely, also applies to DTC. Such hypothesis has not, to the best of our knowledge, been investigated, justifying the usefulness of assessing the effect of combined genotypes on DTC risk.

In the present work we grouped and analysed all studies performed by our group on a Caucasian Portuguese population [14,15,16,17,18]. Since the actual biological situation reflects the concerted action of various alleles in the repair of DNA lesions that may be carcinogenic, all the data was re-analysed in order to identify intra and inter-pathway genotype combinations and thus further characterize the potential contribution of those DNA repair SNPs to DTC susceptibility. Such screening efforts may allow the identification of candidate SNPs for future use as susceptibility biomarkers, hence, the development of tailored DTC prevention policies and perhaps implementation of guidelines.

## 2. Material and Methods

### 2.1. Study Subjects

Overall, 335 Caucasian Portuguese subjects were enrolled in this hospital-based case-control study: 106 histologically confirmed DTC patients were recruited in the Service of Nuclear Medicine of the Portuguese Oncology Institute, Lisbon, Portugal where they were treated according to the hospital current practice and 229 unrelated age (±2 years) and gender-matched controls (two for each DTC case, in each of the previously published studies) were recruited at the Department of Clinical Pathology of the São Francisco Xavier Hospital, West Lisbon Hospital Centre, Portugal where they were seeking healthcare for non-neoplastic pathology. None of the study participants had personal history of prior malignancy nor familial history of thyroid disease.

In order to verify eligibility criteria and to account for potential confounding factors, information on demographic characteristics (e.g., gender, age, occupation), family history of cancer, lifestyle habits (e.g., smoking, alcohol drinking) and IR exposure was collected from each study participant, on recruitment, through a pre-designed questionnaire performed by trained interviewers. Prior exposure to relevant levels of ionizing radiation (i.e., other than that from natural and standard diagnostic sources) was denied by all subjects included in the study. Former smokers were considered as non-smokers if they gave up smoking 2 years before DTC diagnosis or 2 years before their inclusion as controls. The response rate was >95% for both cases and controls. 

All studies were previously approved by the local ethics boards of the involved institutions and conducted in compliance with the Helsinki Declaration. On recruitment, prior to blood withdrawal, all eligible subjects were informed about the objectives of the study. Those agreeing to participate gave their written informed consent and were enrolled in the study. The anonymity of all participants was guaranteed.

### 2.2. SNP Selection

The selection of SNPs for genotyping was performed according to criteria that were predefined individually for each original study [14,15,16,17,18]. Briefly, eligible SNPs were required to exhibit a minor allele frequency (MAF) greater than 0.05 in Caucasian populations, the remaining criteria (e.g., being located in a coding or splice region, altering the amino acid sequence, being a tagging SNP, having been previously referred to in MEDLINE) varying according to the individual study, as indicated in the original studies of individual alleles.

Overall, a total of 36 DNA repair SNPs across all DNA repair pathways were selected for genotyping and analysed. Details on the genomic location, base and amino acid exchange and MAF of selected SNPs are presented on Table 1.

### 2.3. Practical Methodologies—Brief Description

All DNA samples were obtained after collection of peripheral venous blood samples from each participant. The DNA extraction was performed as described previously [14,15,16,17,18] using a commercial available kit (QIAamp^®^ DNA mini kit; Qiagen GmbH, Hilden, Germany), according to the manufacturer’s recommendations. All samples were stored at −20 °C until further analysis.

Genotyping was carried out through either real-time polymerase chain reaction (PCR) or conventional PCR-restriction fragment length polymorphism (RFLP) techniques, as described in previous studies [14,15,16,17,18]. For real-time PCR—the option for the vast majority of SNPs considered in this study – genotyping was performed on an ABI 7300 Real-Time PCR system thermal cycler (Applied Biosystems; Thermo Fisher Scientific, Inc., Waltham, MA, USA), using the commercially available TaqMan^®^ SNP Genotyping Assays (Applied Biosystems) identified in Table 1. Conventional techniques of polymerase chain reaction (PCR) and restriction fragment length polymorphism (RFLP) were employed to genotype *XRCC1* rs1799782, *XRCC1* rs25487 and *OGG1* rs1052133 (BER pathway); *XPC* rs2228000 and *XPC* rs2228001 (NER pathway); and *XRCC3* rs861539 and *XRCC2* rs3218536 (HR pathway). Primer design methods and sequences, PCR conditions, PCR product sizes, restriction analysis conditions and expected digestion pattern for each genotype have been described in full detail elsewhere [14,16,17] and will therefore not be reproduced here. Irrespective of the genotyping method, all inconclusive samples were reanalysed. Also, for quality control, at least 10–15% of genotype determinations were run in duplicates through independent experiments, with 100% concordance between experiments.

### 2.4. Statistical Analysis

Prior to analysis, genotype distributions for each studied SNP were checked for deviation from Hardy–Weinberg equilibrium (HWE) using SNPstat platform [31], in both case and control populations. Variable transformation was applied to categorize the only continuous variable (age of diagnosis) and the Chi-square test was then used to evaluate differences in genotype frequency, smoking status, age class and gender distributions between DTC patients and controls. Whenever the construction of 2 × 2 contingency tables was possible, the two-sided Fisher’s exact test was employed instead of the Chi-square test.

Logistic regression was used to estimate the risk of DTC associated with each genotype: risk estimates were calculated under the codominant, dominant and recessive models and expressed as crude and adjusted odds ratios (OR) and corresponding 95% confidence intervals (CI). Whenever adjustment was performed, terms for gender (male/female), age class (<30, 30–49, 50–69 and ≥70 years) and smoking habits (smokers/non-smokers) were included in the model, the most common homozygous genotype, female gender, lower age group and non-smoking status being considered the reference classes for such calculations. As data on prior IR exposure was not suitable for rigorous quantitative transformation, it was not possible to include such term in the adjustment model. Risk estimates were calculated in the whole population and after stratification according to histological type of tumour (papillary or follicular TC), gender (male and female) and age (<50 and ≥50 years).

Finally, the joint effect of multiple SNPs on DTC risk was estimated from application of logistic regression analysis (1) to relevant haplotypes, (2) to individual genetic risk scores calculated from genotype variables significant on single SNP analysis and (3) to all possible 2 × 2 combinations of the DNA repair SNPs included in this study. For the purpose of risk score calculations, genotypes presenting significant results on single SNP analysis were attributed a +1 score, the risk score for each participant corresponding to the sum of such scores. Samples with one or more missing genotypes were excluded from these calculations to avoid bias due to missing data. For paired SNP analysis, the combination of the most common homozygous genotypes of each individual SNP in the control group was taken as the reference category in OR calculations. Also, paired genotypes with frequency <5% in the study population were pooled together. 

This is not a conclusive final study but an exploratory one that should be regarded as ‘proof of concept’. As such, the Bonferroni adjustment was deemed as not necessary as it is too conservative. Also, the complement of the false negative rate β to compute the power of a test (1−β) was not taken into account at this stage since further studies with more patients and controls should be undertaken to change over this preliminary study into a confirmatory positive one. All statistical analyses were performed with SPSS 22.0 (IBM SPSS Statistics for Windows, version 22.0, IBM Corp, Armonk, NY, USA) except for assessment of HWE deviation, MAF calculations, haplotype estimation and linkage disequilibrium (LD) analysis which were carried out using SNPstats [31]. Results were considered significant when the corresponding two-tailed *p*-values were <0.05 except for paired SNP analysis where, because of the high number of SNP-SNP combinations being tested, a more stringent significance level (*p* < 0.01) was employed. The study was approved by the Ethical Committee of Nova Medical School, Faculty of Medical Sciences with the number 05/2008 dated of January 9th, 2008. The approval was also obtained by the ethical committee of Portuguese Oncology Institute (IPO), the hospital responsible for blood samples collection with the reference GIC/357 dated of July 14th 2004.

## 3. Results

### 3.1. General Analysis 

The general characteristics of the 106 DTC patients and their 229 age- and gender-matched controls included in this study are depicted in Table 2. The overall mean age of the study population was 51 years (52.1 in the patient group and 51.0 in the control group). As expected from the worldwide gender distribution for DTC [1,2], female patients greatly outnumbered male patients in the case group. Twelve (11.3%) DTC patients were categorized as smokers. Age distribution, gender and smoking habits were not significantly different between case and control populations. Concerning histological classification of tumours, 78 (73.6%) patients were diagnosed as papillary TC while 28 (26.4%) presented follicular tumours, in line with DTC histotype distributions commonly reported in the literature [4]. Three additional cases of poorly differentiated TC were also present in some of our original studies but, since this study concerns only with DTC, such cases (and the corresponding controls) were excluded from this analysis. Prior IR exposure (except for diagnostic X-rays) was denied by all cases.

### 3.2. All DTC Cases

Allelic and genotypic frequencies as well as crude/adjusted ORs were calculated for all 36 DNA repair SNPs analysed in our study. Significant findings are reported in Table 3. The allelic and genotypic frequencies observed in the control group were in agreement with those expected for Caucasian populations. Also, for the majority of SNPs, genotype distributions were in Hardy-Weinberg equilibrium (HWE, *p* ≥ 0.05), in both case and control populations. Significant deviations from HWE were observed for *OGG1* rs1052133, *MUTYH* rs3219489 and *CDK7* rs2972388 in the control group and for *XRCC1* rs1799782, *XPC* rs2228000 and *MSH3* rs184967 in the DTC group. Further, strong linkage disequilibrium was observed between *XRCC5* rs1051677 and rs6941, but not between any other pair of SNPs. *XRCC5* rs6941 was thus excluded from further analysis, the conclusions taken for *XRCC5* rs1051677 being valid for *XRCC5* rs6941, since they behave as tag SNPs.

As expected, both the comparison of genotype frequency distributions between case and control populations and the logistic regression analysis (Table 3) yielded results similar to those previously reported [14,15,16,17,18]: significant differences on the distribution of genotypic frequencies between cases and controls were observed for *CCNH* rs2230641 (*p =* 0.037 on the codominant model and *p =* 0.024 on the dominant model), for *MSH6* rs1042821 (*p =* 0.042, on the codominant model and *p =* 0.037 on the recessive model) and for *XRCC3* rs861539 (*p =* 0.021 on the codominant model and *p =* 0.011 on the recessive model). On logistic regression analysis, after adjustment for age, gender and smoking status, DTC risk was significantly increased in *CCNH* rs2230641 heterozygotes (adjusted OR = 1.89, 95% CI: 1.14–3.14, *p =* 0.014) and also in variant allele carriers, according the dominant model (adjusted OR = 1.79, 95% CI: 1.09–2.93, *p =* 0.021), in *MSH6* rs1042821 variant allele homozygotes (adjusted OR = 3.42, 95% CI: 1.04-11.24, *p =* 0.042 on the codominant model; adjusted OR = 3.84, 95% CI: 1.18–12.44, *p =* 0.025 on the recessive model), in *XRCC3* rs861539 variant allele homozygotes (adjusted OR = 2.20, 95% CI: 1.20–4.03, *p =* 0.011 on the recessive model) and in *XPC* rs2228001 variant allele homozygotes (adjusted OR = 1.97, 95% CI: 1.01–3.84, *p =* 0.046 on the recessive model). A borderline significant DTC risk reduction was observed in *ERCC5* rs2227869 heterozygotes (adjusted OR = 0.39, 95% CI: 0.16-1.00, *p =* 0.049). The association between *XPC* rs2228001 and DTC risk is a new finding emerging from this reanalysis, since the recessive model of inheritance had not been applied in the original study [17]. No additional significant differences in genotype frequency distributions nor associations with DTC risk were found, irrespective of the model assumed.

### 3.3. Stratified Analysis

Stratified analysis according to histological tumour type, gender and age may be important to identify any subgroup-specific risk association but was only partially performed in prior studies in this population. On stratification according to histological criteria (Table 4), this study confirmed prior observations [14,17,18] of increased papillary TC risk in *XPC* rs2228001 and *XRCC3* rs861539 variant allele homozygotes (*XPC* rs2228001: adjusted OR = 2.31, 95% CI: 1.07–4.98, *p =* 0.033; *XRCC3* rs861539: adjusted OR = 2.10; 95% CI: 1.07–4.11; *p =* 0.031, both on the recessive model), decreased papillary TC risk in *ERCC5* rs2227869 heterozygotes (adjusted OR = 0.23, 95% CI: 0.07–0.81, *p =* 0.022, on the codominant model) or variant allele carriers (adjusted OR = 0.22, 95% CI: 0.06–0.77, *p =* 0.018, on the dominant model) and increased follicular TC risk in *MLH3* rs175080 variant allele carriers (crude OR = 3.95, 95% CI: 1.05–14.81, *p =* 0.042) and *MSH6* rs1042821 variant allele homozygotes (adjusted OR = 20.98, 95% CI: 1.08-406.53, *p =* 0.044, on the codominant model; adjusted OR = 23.70, 95% CI: 1.25–449.32, *p =* 0.035, on the recessive model). Interestingly, three other significant associations were observed in this reanalysis that were not present or had not been detected in the original studies, while two previously observed associations were lost in this reanalysis: a previously undetected decreased papillary TC risk was observed in *MUTYH* rs3219489 heterozygotes (crude OR = 0.56, 95% CI: 0.32–1.00, *p =* 0.048) and variant allele carriers (crude OR = 0.57, 95% CI: 0.33–0.99, *p =* 0.048) as well as in *NBN* rs1805794 variant allele homozygotes (adjusted OR = 0.28, 95% CI: 0.08-0.97, *p =* 0.045, on the recessive model) while the presence of the variant allele of *XRCC2* rs3218536 exhibited a protective effect for follicular TC (crude OR = 0.21, 95% CI: 0.04–1.00, *p =* 0.049, either for heterozygotes in the codominant model and for variant allele carriers in the dominant model). In contrast, the associations of *XRCC5* rs2440 and *CCNH* rs2230641 genotypes with papillary and follicular TC risk, respectively, reported in our original studies [15,17], were no longer observed. 

On gender stratification (Table 4), when considering female patients only, a significantly increased DTC risk was evident for *CCNH* rs2230641 heterozygotes (adjusted OR = 1.97, 95% CI: 1.13–3.43, *p =* 0.017) and variant allele carriers (adjusted OR = 1.90, 95% CI: 1.11–3.24, *p =* 0.020), for *XPC* rs2228001 variant allele homozygotes (adjusted OR = 2.00, 95% CI: 1.01–3.96, *p =* 0.048, on the recessive model), for *MSH6* rs1042821 variant allele homozygotes (adjusted OR = 4.78, 95% CI: 1.17–19.56, *p =* 0.030, on the codominant model; adjusted OR = 5.42, 95% CI: 1.34–21.92, *p =* 0.018, on the recessive model) and for *XRCC3* rs861539 variant allele homozygotes (adjusted OR = 2.36, 95% CI: 1.12–4.97, *p =* 0.024, on the codominant model; adjusted OR = 2.68, 95% CI: 1.39–5.18, *p =* 0.003, on the recessive model). Opposing, *ERCC5* rs2227869 heterozygotes (adjusted OR = 0.25, 95% CI: 0.07–0.88, *p =* 0.030) and variant allele carriers (adjusted OR = 0.32, 95% CI: 0.11–0.97, *p =* 0.044) as well as *ERCC5* rs17655 variant allele homozygotes (adjusted OR = 0.27, 95% CI: 0.08–0.95, *p =* 0.041, on the recessive model) presented a significant risk reduction among female patients. Among these gender-specific genetic effects, only the association with *MSH6* rs1042821 had been reported in the original studies [18]. No significant association was observed in the male subset of patients, possibly because of the low number of cases in this gender group. An association between *XRCC5* rs1051677 and TC risk had previously been identified in this subset of patients [15] but significance was lost upon restricting analysis to well-differentiated forms of TC (this study). 

Stratified analysis according to the age of diagnosis had only been performed in some of our initial studies, namely those involving SNPs of the BER and MMR pathways [16,18], with negative results. We therefore extended this analysis to the remaining DNA repair SNPs, considering two age groups: <50 and ≥50 years. In patients under 50 years of age, both homozygosity for the *XPC* rs2228001 variant allele (adjusted OR = 2.86, 95% CI: 1.01–8.08, *p =* 0.048, on the recessive model) and the presence of at least one *XRCC5* rs2440 variant allele (adjusted OR = 2.53, 95% CI: 1.02–6.26, *p =* 0.045) were associated with increased DTC risk. When restricting the analysis to patients with 50 or more years of age, DTC risk was increased in *CCNH* rs2230641 heterozygotes (adjusted OR = 2.91, 95% CI: 1.51–5.60, *p =* 0.001) and variant allele carriers (adjusted OR = 3.04, 95% CI: 1.59–5.81, *p =* 0.001), in *RAD51* rs1801321 variant allele homozygotes (adjusted OR = 2.99, 95% CI: 1.25-7.14, *p =* 0.014, on the codominant model; unadjusted OR = 2.03, 95% CI: 1.00–4.12, *p =* 0.049, on the recessive model) and variant allele carriers (adjusted OR = 2.14, 95% CI: 1.06–4.32, *p =* 0.034) and in *XRCC3* rs861539 variant allele homozygotes (adjusted OR = 2.63, 95% CI: 1.16–5.97, *p =* 0.021, on the recessive model). On the contrary, the presence of at least one variant *ERCC6* rs2228529 allele (adjusted OR = 0.47, 95% CI: 0.24–0.92, *p =* 0.028) and its presence in heterozygosity (adjusted OR = 0.48, 95% CI: 0.24–0.97, *p =* 0.042) were associated with a DTC risk reduction in this older age group.

No further correlations between individual DNA repair SNPs and DTC risk were observed on histology-, gender- and age-based stratification analysis.

### 3.4. Combined Genotypes

In order to investigate the joint effect of multiple SNPs on DTC risk, genetic risk scores (RS) were calculated for each study participant, considering only significant findings on single SNP analysis. As depicted in Table 5, after adjusting for covariates, DTC risk was more than two and five times higher in individuals bearing, respectively, 2 (adjusted OR = 2.68, 95% CI: 1.56–4.59, *p* < 0.001) and 3 or more (adjusted OR = 5.02, 95% CI: 2.24–11.24, *p =* 0.001) risk genotypes (*CCNH* rs2230641 Val/Ala or Ala/Ala; *ERCC5* rs2227869 Cys/Cys or Ser/Ser; *XPC* rs2228001 Gln/Gln; *MSH6* rs1042821 Glu/Glu; *XRCC3* rs861539 Met/Met), when compared to individuals bearing none or only one of such risk genotypes. Similar associations between RS and TC risk were also observed on stratification according to histological, gender or age criteria, after adapting RS calculations to the SNPs significant for each strata (Table 5). A high significance level was observed in most cases (*p* < 0.001 in approximately 50% of RS categories) and was even greater if higher RS categories were merged together (results not shown).

**Figure 1:**
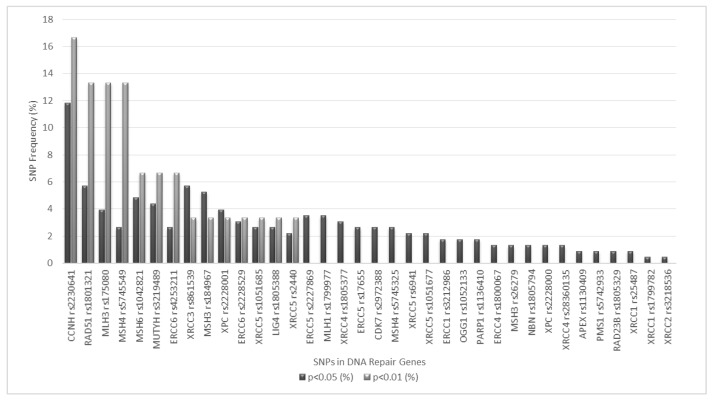
SNP frequency (%) in SNP-SNP pairs showing significant results at *p* < 0.01 and *p* < 0.05 levels. Only SNPs presenting significant results (*p* < 0.05) on combined genotype analysis are shown.

Also, in order to investigate the combined effect of different pairs of SNPs on DTC risk, we performed a paired SNP analysis considering all possible 2 × 2 combinations of the DNA repair SNPs included in this study. Overall, 595 SNP-SNP combinations were tested, 114 (approximately 20%) of which yielded significant results at a 0.05 significance level (results not shown). Considering that such a high number of hypothesis being tested may result in a considerable number of false positive findings, a more stringent significance level (*p* < 0.01) was employed in this analysis, limiting the number of SNP pairs with significant findings to 15 (approximately 2.5% of all possible combinations). Such significant findings are depicted in Table 6 and also in Figure 1. *CCNH* rs2230641 emerges from Figure 1 as the DNA repair SNP most frequently represented in significant SNP-SNP combinations, both at 0.01 and 0.05 significance levels, followed by *RAD51* rs1801321, *MLH3* rs175080 and *MSH4* rs5745549 (0.01 significance level) or *RAD51* rs1801321and *XRCC3* rs861539 (0.05 significance level). MMR variants were the most frequently involved as they were present in 9 of the 15 SNP-SNP combinations that were significant. Also, among significant findings, 3 intra-pathway SNP combinations were detected: *RAD51* rs1801321–*XRCC3* rs861539 (HR pathway), *MLH3* rs175080–*MSH6* rs1042821 and *MSH4* rs5745549–*MSH6* rs1042821 (MMR pathway).

Finally, haplotype analysis was applied to SNPs located in the same chromosome arm, since these are likely to segregate together. According to such criteria, it was possible to establish 8 blocks of DNA repair SNPs, of which only one, located on chromosome 5q and comprising 6 SNPs (*CCNH* rs2230641, *CDK7* rs2972388, *MSH3* rs26279, *MSH3* rs184967, *XRCC4* rs1805377 and *XRCC4* rs28360135), revealed significant associations with DTC (Table 7): two different allele combinations were associated with a significantly decreased DTC risk, when compared to the most frequent combination of chromosome 5q SNPs (adjusted OR1 = 0.26, 95% CI: 0.08–0.87, *p =* 0.030; adjusted OR2 = 0.15, 95% CI: 0.03–0.72, *p =* 0.019). Haplogroup analysis comprising all SNPs under study could also prove useful to understand the joint effect of the variants since it would better reflect the real context situation (where different DNA repair proteins interact with each other) but could not be performed because, considering the high number of SNPs under study, the frequency of each specific allele combination would be too low for meaningful results to be obtained.

## 4. Discussion

In order to further characterize the potential contribution of DNA repair SNPs to DTC susceptibility, we aggregated and reanalysed the data from our previously published case-control studies [14,15,16,17,18] performed on a Caucasian Portuguese population. 

A significant risk increase was observed, after adjustment for age, gender and smoking status, in *CCNH* rs2230641 heterozygotes and variant allele carriers, in *MSH6* rs1042821 variant allele homozygotes (codominant and recessive model), in *XRCC3* rs861539 variant allele homozygotes (recessive model) and in *XPC* rs2228001 variant allele homozygotes (recessive model), while the heterozygous *ERCC5* rs2227869 genotype was associated with a borderline risk reduction. Except for *XPC* rs2228001, which is a new finding emerging from this reanalysis because the recessive model of inheritance had not been applied in the original study, such results are fundamentally similar to those reported on the original studies despite, on reanalysis, data was restricted to DTC cases and corresponding controls. A role for these variants specifically on well-differentiated forms of TC is thus apparent from this reanalysis. As these findings have been discussed in detail in the original studies, they will be discussed here only briefly, with emphasis on new data published since then.

XRCC3 participates in HR to maintain chromosome stability and repair DNA damage and is therefore a highly suspected candidate gene for cancer susceptibility. The *XRCC3* rs861539 has been the most studied genetic variant of *XRCC3* gene, especially because is located in a functional relevant domain of the protein, in an interaction region with other proteins such as RAD51 [22,32]. The presence of this variant may affect the structure of this DNA repair protein and lead to a deficiency in the HR pathway. As a result, the HR pathway may be compromised, shifting the repair mechanism to NHEJ, promoting chromosome instability and disturbing the cellular repair capacity [33]. The potential contribution of *XRCC3* rs861539 to cancer susceptibility has been widely addressed: while conflicting evidence exists, several large meta-analyses strongly support a positive association with cancer susceptibility, namely breast [34,35,36] and bladder cancer [36,37,38], among others. In the particular context of thyroid cancer, interestingly, multiple studies [22,39,40,41,42,43], including a meta-analysis [44], have suggested the *XRCC3* rs861539 variant T allele and/or, in particular, the TT homozygous genotype to be associated with increased risk of TC or, more specifically, PTC. In another meta-analysis [45] such association was also detected but only in Caucasian populations. Therefore, despite studies reporting no significant association also exist [46,47], the vast majority of available evidence supports our results and suggests a role for *XRCC3* rs861539 in DTC susceptibility. 

To the best of our knowledge, none of the remaining SNPs presenting significant results on overall analysis has been evaluated in the context of DTC (or TC) susceptibility.

*XPC* codes for a DNA binding protein that acts forming the distortion-sensing component of NER by binding tightly with another important NER protein, HR23B, to form a stable XPC-HR23B complex, thus playing a central role in the process of early damage recognition [48,49]. XPC-HR23B complex can recognize a variety of DNA adducts formed by exogenous carcinogens and binds to the DNA damage sites. Therefore, it may play a role in decreasing the toxic effects of such carcinogens and its deficiency may interact with carcinogen exposure [50]. XPC is also involved in DNA damage-induced cell cycle checkpoint regulation and apoptosis, removal of oxidative DNA damage and redox homeostasis [49,51]. *XPC* rs2228001 (an A-to-C transition in exon 15) leads to a substitution of glutamine for lysine in codon 939 (Lys939Gln) and is located in the domain interacting with the transcription factor IIH (TFIIH) complex [50,52,53,54,55], initiating the global genome NER pathway. *XPC* rs2228001 is one of the most extensively studied NER pathway SNPs, as numerous case-control association studies and meta-analyses have been performed to investigate its potential role on cancer predisposition. In line with our data for DTC, a modest but consistent association of the Gln/Gln homozygous genotype with overall cancer risk is apparent from two of the three meta-analysis that pool data from different cancer types [56,57,58]. Evidence from these and other cancer site-specific meta-analyses is stronger for lung [53,56,57,58,59,60], bladder [54,56,61,62] and colorectal cancer (CRC) [56,58] [63,64], but also exists for other cancer types such as upper digestive system cancer [65] and hepatocellular carcinoma [50,66]. *XPC* rs2228001 genotype has also been found to correlate with survival of hepatocellular patients [66], with *XPC* mRNA expression levels [60,66,67], with drug-induced toxicity in cancer patients treated with platinum-based chemotherapeutic agents (e.g., cisplatin) [68,69], with sensitivity of lung squamous cell carcinoma patients to chemotherapy [67] and to interfere with the capacity to repair DNA lesions induced by, e.g., benzo(a)pyrene [70,71,72], gamma-radiation [70], X-rays [73], UV radiation [74], aflatoxin B1 [50] and meat-derived carcinogens [75]. Overall, evidence strongly suggests that *XPC* rs2228001 genotype is associated with altered DNA repair capacity, establishing ground for a putative role of this SNP in cancer susceptibility.

The *MSH6* gene (mutS homolog 6) is a member of a set of genes known as the mismatch repair (MMR) genes. MSH6 integrates the MutSα complex, a sensor of genetic damage that, besides its role in the repair of replication errors, cooperates with other DNA repair and damage-response signalling pathways to allow for cell cycle arrest, DNA repair and/or apoptosis of genetically damaged cells. Several *MSH6* mutations have been identified and suggested as causative in Lynch syndrome (LS) patients [76,77,78,79,80]. Despite TC is not part of the usual LS spectrum, the effect of *MSH6* in TC susceptibility has previously been explored [81,82]. *MSH6* rs1042821 has also been frequently investigated in the context of cancer susceptibility, mostly with inconclusive findings [83,84,85,86,87,88,89,90]. Consistent with our results, *MSH6* rs1042821 has previously been associated with increased CRC risk [91,92,93], highly malignant bladder cancer [94], pancreatic cancer [95] and triple negative breast cancer (TNBC) [96]. On the contrary, the T allele [97] and the CT heterozygous genotype [98] have been associated with decreased colorectal and hepatocellular carcinoma, respectively. The only meta-analysis concerning the role of *MSH6* rs1042821 on cancer predisposition that we are aware of is also inconclusive [99]. Despite plausible, a potential role for *MSH6* rs1042821 on cancer predisposition (DTC, in particular) remains elusive. Further well-powered studies are needed to clarify this issue.

The role of *CCNH* rs2230641 on cancer predisposition has only seldom been evaluated: in agreement with our results, a significantly increased bladder cancer risk in ever smokers has been reported for C allele carriers [100] but, on the contrary, such genotype has also been associated with a significantly decreased risk of chronic leukaemia [101]. Most other studies, namely in oesophageal [102], bladder [103], biliary tract [104] and renal cell carcinoma [105], as well as in oral premalignant lesions [106] have been inconclusive. Interestingly, the pharmacogenomic implications of *CCNH* rs2230641 on the outcome of platinum-based chemotherapy have also been evaluated, results supporting a role for *CCNH* rs2230641 on the response to DNA damaging agents: the presence of the *CCNH* rs2230641 variant C allele has been associated with longer survival in NLCSC patients receiving platinum-based chemotherapy [107] and with increased incidence and severity of oxaliplatin-induced acute peripheral neuropathy in digestive tract cancer patients undergoing with the oxaliplatin-based chemotherapy [108]. Similarly, increased risk of severe oxaliplatin-induced acute peripheral neuropathy was observed by Custodio et al. [109] in high-risk stage II and stage III colon cancer patients homozygous for the C allele, submitted to oxaliplatin-based adjuvant chemotherapy. *CCNH* codes for a highly conserved cyclin protein that participates in several cellular processes such as the NER pathway, cell cycle regulation and receptor phosphorylation, among others [48,110]. Although data on the functional relevance of rs2230641 is lacking, the pleiotropic effects of CCNH confer biological plausibility to our hypothesis that *CCNH* variants may be involved in cancer susceptibility.

Finally, *ERCC5*, also known as *XPG*, is located on chromosome 13q22–q33 [111] and comprises 15 exons [112,113]. It encodes a structure-specific endonuclease that has multiple functions during NER [114], reason why defects in this gene can impair DNA repair resulting in genomic instability and carcinogenesis [115]. In fact, only a few studies have considered the putative contribution of *ERCC5* rs2227869 to cancer susceptibility, most being inconclusive. Interestingly, the only significant findings reported thus far are in line with those reported here, suggesting a protective role for the heterozygous genotype: Hussain et al. [116] reported a significant reduction in stomach cancer risk in heterozygous genotype individuals and a similar, despite nonsignificant, trend has also been independently observed for melanoma [117] and for squamous cell carcinoma of the head and neck (SCCHN) [118]. More importantly, in the only meta-analysis performed to date [119], a decrease in cancer risk in *ERCC5* rs2227869 heterozygotes (and for the C allele) has also been reported. 

Many of these (and other) SNPs also presented significant findings on stratifying data according to hystotype, gender and age: on histological stratification, significant associations were observed between *XRCC3* rs861539, *XPC* rs2228001, *ERCC5* rs2227869, *MUTYH* rs3219489 and *NBN* rs1805794 and papillary TC, while *MSH6* rs1042821, *MLH3* rs175080 and *XRCC2* rs3218536 were associated with follicular TC. *XRCC3* rs861539, *XPC* rs2228001, *MSH6* rs1042821, *CCNH* rs2230641, *ERCC5* rs2227869 and *ERCC5* rs17655 were associated with DTC in the female subset while no association was observed in males. Finally, *XPC* rs2228001 and *XRCC5* rs2440 were associated with DTC in participants younger than 50 years, while, in participants aged 50 or more years, the DTC-associated SNPs included *XRCC3* rs861539, *CCNH* rs2230641, *ERCC6* rs2228529 and *RAD51* rs1801321.

It is unclear whether these findings (and which among these) truly represent group-specific effects or whether they simply reflect the overall effect on the largest groups (i.e., when group sizes are unbalanced, e.g., papillary TC vs follicular TC, female vs male) and the corresponding lack of power to detect an effect on the smallest groups. Also, due to the low sample size on each strata, some of these results may simply represent incident findings (type I errors). *XRCC3* rs861539, for example, has been previously associated with papillary TC [22,39,40]—in line with our results—but not with follicular TC. An effect of *XRCC3* rs861539 genotype in follicular TC cannot, however, be excluded since follicular TC is much less frequent than papillary TC and these studies may have been underpowered to detect such effect. Also, Su et al. [120] have demonstrated the homozygous genotype of this SNP to be associated with breast cancer, the association being stronger in women younger than 55 years, with earlier first menarche or with latter menopause. This suggests an oestrogen-potentiated genetic effect, compatible with our own observation of increased DTC risk in *XRCC3* rs861539 TT homozygotes among females but not among males. Further, the involvement of CCNH, through a cyclin-activated kinase complex, in oestrogen receptor phosphorylation [48] provides a possible rationale for our own observation of an association of the *CCNH* rs2230641genotype with DTC among females but not among males. Finally, the association of *MSH6* rs1042821with DTC, observed in this study for female but not male individuals, is compatible with the growing evidence placing DTC as an oestrogen-associated cancer [121,122,123,124] and implicating *MSH6* in such cancers [78,125,126,127,128,129]. These selected examples highlight the plausibility of the existence of group-specific genetic effects. Overall, such hystotype, gender and age specifies in DTC susceptibility are likely since (1) papillary and follicular TC represent distinct entities, with hystotype-specific molecular profiles (e.g., *BRAF* mutations and *RET*/*PTC* rearrangements in PTC, *RAS* mutations and *PAX8*/*PPARγ* translocations in FTC) [130]; (2) important gender differences exist in the incidence of DTC (i.e., DTC is, as previously stated two to four times more frequent in women than in men) [1,2]; and (3) DTC presents some age specificities, uncommon in other types of cancer (DTC is one of the most common malignancies in adolescent and young adults, the median age at diagnosis being lower than that for most other types of cancer) [1,2]. Further well-powered studies are urgently needed to clarify these results and thus establish which of these SNPs, if any, represents true group-specific susceptibility biomarkers.

Considering the multifactorial nature of DTC aetiology and the probable involvement of multiple genetic factors, alone or in combination, in DTC susceptibility, we undertook a combined genotype analyses to investigate the joint effect of multiple SNPs on DTC risk. When combining all risk genotypes significant at single SNP analysis into a unique unbalanced risk score, a clear-cut gene-dosage effect between the number of risk genotypes (unbalanced risk score) and DTC risk was observed, both on global analysis (considering all DTC cases and corresponding controls) and after stratification according to histological, gender and age criteria. This is biologically plausible since the different DNA repair proteins physically and functionally interact with each other, within the same or different DNA repair pathways, establishing ground for additive or even multiplicative effects of different SNPs on DNA repair activity and, hence, cancer risk. Such polygenic approach to assess the cumulative effects of multiple genetic variants on cancer risk has previously been employed [27,107,131,132], supporting its usefulness and clinical potential.

To investigate the effect of specific DNA repair SNP combinations on DTC risk, all possible 2 × 2 combinations were tested on paired SNP analysis, yielding fifteen SNP pairs with *p* < 0.01. Multiple interactions between SNPs from different DNA repair pathways and, even, other DNA damage response proteins have previously been reported [39,42,66,87], providing a rationale for such approach. Of notice, *CCNH* rs2230641 was the most frequently represented DNA repair SNP in such significant combinations, both at 0.01 and 0.05 significance levels, a finding that is compatible with the pleiotropic role of CCNH in DNA damage repair, cell cycle regulation and receptor phosphorylation [48,110]. More importantly, the contribution of MMR variants to the joint effect of DNA repair SNPs on DTC risk is evident from our results, as they were present in 9 of the 15 SNP pairs presenting significant findings. Besides its critical role in post-replication repair (through recognition and repair of base-base mispairs and insertion/deletion loops that arise during replication), the MMR pathway cooperates with other repair pathways in the recognition and subsequent repair of DNA damage induced by IR, UV light, oxidative stress or genotoxic chemicals (e.g., oxidative lesions, double strand breaks, pyrimidine dimers and inter-strand crosslinks) and contributes to damage-induced cytotoxicity through downstream signalling for cell cycle arrest and apoptosis [133,134,135]. Therefore, considering the large spectre of action of the MMR pathway, an elevated number of interactions between MMR and other DNA repair SNPs is expected. Such hypothesis, in line with our findings, has been recently strengthened by a report [136] associating SNPs from different DNA repair pathways with CRC in Lynch syndrome patients, a cancer predisposition condition originated by germline MMR mutations. Finally, among SNP pairs presenting significant findings in this study, three are intra-pathway combinations involving either HR or MMR pathway SNPs. The joint effects of *MLH3* rs175080 – *MSH6* rs1042821 and *MSH4* rs5745549 – *MSH6* rs1042821 (MMR pathway) SNP combinations were reported and discussed in our original study [18]. The joint effect of *RAD51* rs1801321 and *XRCC3* rs861539 (HR pathway) on cancer risk has been previously reported for breast cancer [137], in line with our results, and may be of particular relevance for DTC since the formation of radiation damage-induced RAD51 foci requires functional XRCC3 [138].

Finally, on applying haplotype analysis to SNPs that are located in the same chromosome arm (thus likely to segregate together), one block of DNA repair SNPs located on chromosome 5q (comprising *CCNH* rs2230641, *CDK7* rs2972388, *MSH3* rs26279, *MSH3* rs184967, *XRCC4* rs1805377 and *XRCC4* rs28360135) was associated with DTC risk in our study. Such results further suggest an independent or interactive effect of these SNPs on DTC predisposition.

Overall, our results suggest that DNA repair SNPs across different pathways and may contribute to DTC predisposition, possibly exerting cumulative effects. This is of relevance since the estimated high heritability of DTC is only partially explained, even when considering the contribution of several GWAS recently performed. Gene-gene and gene-environment interactions have been hypothesised to play an important role so their identification and in-depth study is highly desirable to explain the “missing” heritability of DTC. However, the results presented here should be regarded only as proof of concept and must therefore be validated through replication in larger independent populations. Future studies should also be designed with the intention of accounting for environmental factors such as IR exposure and iodine deficiency (and their potential interaction with genetic factors). In addition, they should be sufficiently powered to allow other, less frequent but potentially relevant SNPs, to be studied and to allow more sophisticated and conclusive gene-gene interaction analysis to be performed. Finally, in order to strengthen our preliminary findings, the functional significance of these SNPs should be further investigated as well as their potential association with mutational events involved in DTC carcinogenesis (e.g., *BRAF* mutations and *RET*/*PTC* rearrangements).

## Figures and Tables

**Table 1:** Selected SNPs and detailed information on the corresponding base and amino acid exchanges, minor allele frequency (MAF) and AB assay used for genotyping.

Gene	Location	db SNP Cluster ID (rs no.)	Base Change	Aminoacid Change	MAF (%) ^a^	AB Assay ID
**Base Excision Repair (BER)**						
*XRCC1*	19q13.31	rs1799782	C → T	Arg194Trp	13.1	--^e^
	19q13.31	rs25487	G → A	Arg399Gln	26.6	--^e^
*OGG1*	3p25.3	rs1052133	C → G	Ser326Cys	29.9	--^e^
*APEX1*	14q11.2	rs1130409	T → G	Asp148Glu	44.0	C___8921503_10
*MUTYH*	1p34.1	rs3219489	G → C	Gln335His	31.9	C__27504565_10
*PARP1*	1q42.12	rs1136410	T → C	Val762Ala	24.4	C___1515368_1_
**Nucleotide Excision Repair (NER)**						
*CCNH*	5q14.3	rs2230641	T → C	Val270Ala	13.8	C__11685807_10
*CDK7*	5q13.2	rs2972388	A → G	Asn33Asn	40.5	C___1191757_10
*ERCC5*	13q33.1	rs2227869	G → C	Cys529Ser	4.9	C__15956775_10
	13q33.1	rs17655	C → G	Asp1104His	37.7	C___1891743_10
*ERCC1*	19q13.32	rs3212986	G → T	-- ^b^	29.4	C___2532948_10
*RAD23B*	9q31.2	rs1805329	C → T	Ala249Val	16.7	C__11493966_10
*ERCC6*	10q11.23	rs2228529	A → G	Gln1413Arg	15.6	C__16171343_10
	10q11.23	rs4253211	G → C	Arg1230Pro	6.4	C__25762749_10
*ERCC4*	16p13.12	rs1800067	G → A	Arg415Gln	3.1	C___3285104_10
*XPC*	3p25.1	rs2228000	C→T	Ala499Val	24.8	--^e^
	3p25.1	rs2228001	A→C	Lys939Gln	34.4	--^e^
**Mismatch Repair (MMR)**						
*MLH1*	3p22.2	rs1799977	A → G	Ile219Val	13.0	C___1219076_20
*MSH3*	5q14.1	rs26279	A → G	Thr1045Ala	28.0	C____800002_1_
	5q14.1	rs184967	G → A	Arg949Gln	9.8	C____907914_10
*MSH4*	1p31.1	rs5745549	G → A	Ser914Asn	6.4	C___1184803_10
	1p31.1	rs5745325	G → A	Ala97Thr	21.3	C___3286081_10
*PMS1*	2q32.2	rs5742933	G → C	-- ^c^	21.9	C__29329633_10
*MLH3*	14q24.3	rs175080	G → A	Pro844Leu	36.4	C___1082805_10
*MSH6*	2p16.3	rs1042821	C → T	Gly39Glu	20.1	C___8760558_10
**Homologous Recombination (HR)**						
*RAD51*	15q15.1	rs1801321	G → T	-- ^c^	25.7	C___7482700_10
*NBN*	8q21.3	rs1805794	C → G	Glu185Gln	35.7	C__26470398_30
*XRCC2*	7q36.1	rs3218536	G → A	Arg188His	5.3	--^e^
*XRCC3*	14q32.33	rs861539	C → T	Thr241Met	21.7	--^e^
**Non-homologous End Joining (NHEJ)**						
*XRCC4*	5q14.2	rs1805377	G → A	-- ^d^	37.5	C__11685997_10
*LIG4*	13q33.3	rs1805388	C → T	Thr9Ile	14.6	C__11427969_20
*XRCC4*	5q14.2	rs28360135	T → C	Ile134Thr	1.4	C__25618660_10
*XRCC5*	2q35	rs1051685	A → G	-- ^b^	17.2	C___8838368_1_
	2q35	rs1051677	T → C	-- ^b^	15.6	C___8838367_1_
	2q35	rs6941	C → A	-- ^b^	15.7	C___8838374_10
	2q35	rs2440	C → T	-- ^b^	42.0	C___3231046_10

^a^ Minor Allele Frequency, according to http://www.ncbi.nlm.nih.gov/projects/SNP/. ^b^ SNP located on 3’ UTR. ^c^ SNP located on 5’ UTR. ^d^ SNP located on intron. ^e^ not applicable (genotyping performed by PCR-RFLP). SNPs, single nucleotide polymorphisms.

**Table 2:** General characteristics for the DTC case (*n* = 106) and control (*n* = 229) populations.

Characteristics		Controls *n* (%)	Cases *n* (%)	*p*-Value ^c^
**Gender**	Male	43 (18.8)	16 (15.1)	0.445
	Female	186 (81.2)	90 (84.9)	
**Age ^a, b^**	<30	14 (6.1)	4 (3.8)	0.817
	30–49	85 (37.1)	38 (35.8)	
	50–69	100 (43.7)	49 (46.2)	
	≥70	30 (13.1)	15 (14.2)	
**Smoking habits**	Non-smokers	184 (80.3)	94 (88.7)	0.084
	Smokers	43 (18.8)	12 (11.3)	
	Missing	2 (0.9)	0 (0.0)	

^a^ Age of diagnosis, for cases. ^b^ Age at the time of diagnosis of the matched case, for controls. ^c^ *p*-value for cases versus control group determined by two-sided Fisher’s exact test (gender, smoking habits) or χ^2^ test (age). Abbreviations: DTC, well-differentiated thyroid cancer.

**Table 3:** Genotype distribution in case and control populations and associated DTC risk (crude and adjusted ORs). Only SNPs presenting significant findings are shown.

Genotype	MAF		Genotype Frequency		*p*-Value ^a^	OR (95% CI)	Adjusted OR (95% CI) ^b^
	Controls	Cases	Controls n (%)	Cases n (%)			
***CCNH*** **rs2230641**			212 (100)	106 (100)			
Val/Val	C: 0.17	C: 0.23	148 (69.8)	60 (56.6)	**0.037 ^c^**	1 (Reference)	1 (Reference)
Val/Ala			56 (26.4)	43 (40.6)		**1.89 (1.15–3.12) ^c^**	**1.89 (1.14–3.14) ^c^**
Ala/Ala			8 (3.8)	3 (2.8)		0.93 (0.24–3.61)	1.01 (0.25-4.04)
Dominant model			64 (30.2)	46 (43.4)	**0.024 ^c^**	**1.77 (1.09–2.87) ^c^**	**1.79 (1.09–2.93) ^c^**
Recessive model			8 (3.8)	3 (2.8)	0.757	0.74 (0.19–2.86)	0.80 (0.20–3.17)
***ERCC5* rs2227869**			212 (100)	106 (100)			
Cys/Cys	C: 0.07	C: 0.04	184 (86.8)	99 (93.4)	0.135	1 (Reference)	1 (Reference)
Cys/Ser			27 (12.7)	6 (5.7)		0.41 (0.17–1.03)	**0.39 (0.16–1.00) ^c^**
Ser/Ser			1 (0.5)	1 (0.9)		1.86 (0.12–30.04)	1.78 (0.11–29.13)
Dominant model			28 (13.2)	7 (6.6)	0.088	0.47 (0.20–1.10)	0.44 (0.19–1.06)
Recessive model			1 (0.5)	1 (0.9)	1.000	2.01 (0.12–32.45)	1.92 (0.12–31.48)
***XPC*** **rs2228001**			212 (100)	106 (100)			
Lys/Lys	C: 0.36	C: 0.41	82 (38.7)	39 (36.8)	0.103	1 (Reference)	1 (Reference)
Lys/Gln			108 (50.9)	47 (44.3)		0.92 (0.55–1.53)	0.95 (0.57–1.60)
Gln/Gln			22 (10.4)	20 (18.9)		1.91 (0.94–3.91)	1.92 (0.93–3.97)
Dominant model			130 (61.3)	67 (63.2)	0.807	1.08 (0.67–1.76)	1.12 (0.69–1.82)
Recessive model			22 (10.4)	20 (18.9)	0.052	**2.01 (1.04–3.87) ^c^**	**1.97 (1.01–3.84) ^c^**
***MSH6* rs1042821**			210 (100)	106 (100)			
Gly/Gly	T: 0.21	T: 0.22	127 (60.5)	68 (64.2)	**0.042 ^c^**	1 (Reference)	1 (Reference)
Gly/Glu			78 (37.1)	30 (28.3)		0.72 (0.43–1.20)	0.73 (0.43–1.23)
Glu/Glu			5 (2.4)	8 (7.5)		2.99 (0.94–9.49)	**3.42 (1.04–11.24) ^c^**
Dominant model			83 (39.5)	38 (35.8)	0.543	0.86 (0.53–1.39)	0.87 (0.54–1.43)
Recessive model			5 (2.4)	8 (7.5)	**0.037 ^c^**	**3.35 (1.07–10.50) ^c^**	**3.84 (1.18–12.44) ^c^**
***XRCC3* rs861539**			209 (100)	106 (100)			
Thr/Thr	T: 0.40	T: 0.45	70 (33.5)	36 (34.0)	**0.021 ^c^**	1 (Reference)	1 (Reference)
Thr/Met			112 (53.6)	44 (41.5)		0.76 (0.45–1.30)	0.77 (0.45–1.31)
Met/Met			27 (12.9)	26 (24.5)		1.87 (0.96–3.67)	1.89 (0.96–3.72)
Dominant model			139 (66.5)	70 (66.0)	1.000	0.98 (0.60–1.61)	0.99 (0.60–1.62)
Recessive model			27 (12.9)	26 (24.5)	0.011 ^c^	**2.19 (1.20–3.99) ^c^**	**2.20 (1.20–4.03) ^c^**

^a^*p*-value for cases versus control group determined by two-sided Fisher’s exact test (whenever 2 × 2 contingency tables are possible) or χ^2^ test (remaining cases). ^ b^ ORs were adjusted for gender (male and female), age (<30, 30–49, 50-69, ≥ 70 years) and smoking status (non-smoker and smoker). ^c^ *p* < 0.05. Abbreviations: DTC, well-differentiated thyroid cancer; MAF, minor allele frequency; OR, odds ratio; CI, confidence interval.

**Table 4:** Genotype distribution in the case population (*n* = 106) and associated DTC risk (crude and adjusted ORs), after stratification according to histological type, gender and age. Only SNPs presenting significant findings are shown.

**Genotype**	**Papillary Carcinoma**			**Follicular Carcinoma**		
	***n*** **(%)**	**Crude OR** **(95% CI)**	**Adjusted OR** **(95% CI) ^a^**	***n*** **(%)**	**Crude OR** **(95% CI)**	**Adjusted OR** **(95% CI) ^a^**
***MUTYH*** **rs3219489**	78 (100)			28 (100)		
Gln/Gln	48 (61.5)	1 (reference)	1 (reference)	15 (53.6)	1 (reference)	1 (reference)
Gln/His	27 (34.6)	**0.56 (0.32** **–** **1.00) ^b^**	0.57 (0.32–1.02)	11 (39.3)	0.95 (0.37–2.43)	1.09 (0.40–2.92)
His/His	3 (3.8)	0.66 (0.16–2.68)	0.69 (0.17–2.86)	2 (7.1)	4.13 (0.35–49.28)	6.97 (0.47–104.26)
Dominant model	30 (38.5)	**0.57 (0.33–0.99) ^b^**	0.58 (0.33–1.02)	13 (46.4)	1.08 (0.43–2.67)	1.27 (0.49–3.29)
Recessive model	3 (3.8)	0.85 (0.21–3.36)	0.87 (0.22–3.54)	2 (7.1)	4.23 (0.37–48.8)	6.75 (0.46–98.39)
***ERCC5* rs2227869**	78 (100)			28 (100)		
Cys/Cys	75 (96.2)	1 (reference)	1 (reference)	24 (85.7)	1 (reference)	1 (reference)
Cys/Ser	3 (3.8)	**0.24 (0.07–0.84) ^b^**	**0.23 (0.07–0.81) ^b^**	3 (10.7)	1.28 (0.28–5.78)	1.20 (0.26–5.61)
Ser/Ser	0 (0.0)	--	--	1 (3.6)	--	--
Dominant model	3 (3.8)	**0.23 (0.07–0.80) ^b^**	**0.22 (0.06–0.77) ^b^**	4 (14.3)	1.70 (0.42–6.90)	1.61 (0.38–6.74)
Recessive model	0 (0.0)	--	--	1 (3.6)	--	--
***XPC*** **rs2228001**	78 (100)			28 (100)		
Lys/Lys	26 (33.3)	1 (reference)	1 (reference)	13 (46.4)	1 (reference)	1 (reference)
Lys/Gln	36 (46.2)	1.01 (0.55–1.85)	1.03 (0.56–1.90)	11 (39.3)	0.72 (0.27–1.91)	0.91 (0.33–2.54)
Gln/Gln	16 (20.5)	2.27 (0.99–5.22)	2.35 (1.00–5.51)	4 (14.3)	1.18 (0.28–4.96)	1.05 (0.24–4.65)
Dominant model	52 (66.7)	1.22 (0.69–2.15)	1.23 (0.69–2.20)	15 (53.6)	0.80 (0.32–2.01)	0.94 (0.36–2.44)
Recessive model	16 (20.5)	**2.26 (1.06–4.80) ^b^**	**2.31 (1.07–4.98) ^b^**	4 (14.3)	1.39 (0.36–5.39)	1.10 (0.27–4.51)
***MLH3*** **rs175080**						
Pro/Pro	19 (24.4)	1 (reference)	1 (reference)	3 (10.7)	1 (reference)	1 (reference)
Pro/Leu	42 (53.8)	1.13 (0.59–2.19)	1.17 (0.60–2.27)	17 (60.7)	3.78 (0.97–14.79)	3.61 (0.88–14.85)
Leu/Leu	17 (21.8)	1.17 (0.53–2.61)	1.20 (0.54–2.68)	8 (28.6)	4.36 (0.95–20.04)	4.29 (0.89–20.78)
Dominant model	59 (75.6)	1.14 (0.61–2.14)	1.18 (0.62–2.22)	25 (89.3)	**3.95 (1.05–14.81) ^b^**	3.81 (0.97–14.95)
Recessive model	17 (21.8)	1.08 (0.56–2.10)	1.08 (0.56–2.10)	8 (28.6)	1.64 (0.57–4.69)	1.67 (0.55–5.02)
***MSH6* rs1042821**	78 (100)			28 (100)		
Gly/Gly	49 (62.8)	1 (reference)	1 (reference)	19 (67.9)	1 (reference)	1 (reference)
Gly/Glu	24 (30.8)	0.74 (0.41–1.32)	0.74 (0.41–1.35)	6 (21.4)	0.65 (0.22–1.91)	0.76 (0.24–2.35)
Glu/Glu	5 (6.4)	2.30 (0.59–8.95)	2.47 (0.61–9.89)	3 (10.7)	5.84 (0.57–60.03)	**20.98 (1.08–406.53) ^b^**
Dominant model	29 (37.2)	0.83 (0.48–1.46)	0.85 (0.48–1.49)	9 (32.1)	0.92 (0.35–2.43)	1.10 (0.39–3.07)
Recessive model	5 (6.4)	2.57 (0.67–9.85)	2.74 (0.69–10.84)	3 (10.7)	6.60 (0.65–66.63)	**23.70 (1.25–449.32) ^b^**
***NBN*** **rs1805794**	78 (100)			28 (100)		
Glu/Glu	42 (53.8)	1 (reference)	1 (reference)	13 (46.4)	1 (reference)	1 (reference)
Glu/Gln	33 (42.3)	1.17 (0.66–2.07)	1.15 (0.64–2.04)	10 (35.7)	0.90 (0.33–2.41)	0.72 (0.25–2.05)
Gln/Gln	3 (3.8)	0.31 (0.09–1.10)	0.29 (0.08–1.06)	5 (17.9)	2.69 (0.62–11.71)	2.23 (0.44–11.18)
Dominant model	36 (46.2)	0.95 (0.55–1.64)	0.94 (0.54–1.63)	15 (53.6)	1.15 (0.47–2.86)	0.90 (0.34–2.39)
Recessive model	3 (3.8)	0.29 (0.08–1.01)	**0.28 (0.08–0.97) ^b^**	5 (17.9)	2.83 (0.70–11.50)	2.66 (0.58–12.06)
***XRCC2*** **rs3218536**	78 (100)			28 (100)		
Arg/Arg	66 (84.6)	1 (reference)	1 (reference)	26 (92.9)	1 (reference)	1 (reference)
Arg/His	12 (15.4)	1.17 (0.54–2.52)	1.19 (0.55–2.57)	2 (7.1)	**0.21 (0.04–1.00) ^b^**	0.20 (0.04–1.05)
His/His	0 (0.0)	--	--	0 (0.0)	--	--
Dominant model	12 (15.4)	1.17 (0.54–2.52)	1.19 (0.55–2.57)	2 (7.1)	**0.21 (0.04–1.00) ^b^**	0.20 (0.04–1.05)
Recessive model	0 (0.0)	--	--	0 (0.0)	--	--
***XRCC3*** **rs861539**	78 (100)			28 (100)		
Thr/Thr	26 (33.3)	1 (reference)	1 (reference)	10 (35.7)	1 (reference)	1 (reference)
Thr/Met	31 (39.7)	0.75 (0.40–1.40)	0.74 (0.39–1.39)	13 (46.4)	0.81 (0.30–2.20)	0.78 (0.27–2.24)
Met/Met	21 (26.9)	1.76 (0.82–3.75)	1.76 (0.82–3.77)	5 (17.9)	2.50 (0.55–11.41)	2.72 (0.54–13.60)
Dominant model	52 (66.7)	0.97 (0.54–1.73)	0.97 (0.54–1.73)	18 (64.3)	1.00 (0.39–2.58)	1.00 (0.37–2.69)
Recessive model	21 (26.9)	**2.08 (1.07–4.06) ^b^**	**2.10 (1.07–4.11) ^b^**	5 (17.9)	2.83 (0.70–11.50)	3.12 (0.69–14.02)
						
**Genotype**	**Male**			**Female**		
	***n*** **(%)**	**OR (95% CI)**	**Adjusted OR (95% CI) ^a^**	***n*** **(%)**	**OR (95% CI)**	**Adjusted OR (95% CI) ^a^**
***CCNH* rs2230641**	16 (100)			90 (100)		
Val/Val	7 (43.8)	1 (reference)	1 (reference)	53 (58.9)	1 (reference)	1 (reference)
Val/Ala	9 (56.3)	1.38 (0.40–4.70)	1.67 (0.44–6.34)	34 (37.8)	**2.03 (1.17–3.53) ^b^**	**1.97 (1.13–3.43) ^b^**
Ala/Ala	0 (0.0)	--	--	3 (3.3)	1.26 (0.30–5.20)	1.36 (0.32–5.78)
Dominant model	9 (56.3)	1.21 (0.36–4.06)	1.40 (0.38–5.17)	37 (41.1)	**1.93 (1.13–3.30) ^b^**	**1.90 (1.11–3.24) ^b^**
Recessive model	0 (0.0)	--	--	3 (3.3)	1.01 (0.25–4.12)	1.11 (0.26–4.68)
***ERCC5* rs2227869**	16 (100)			90 (100)		
Cys/Cys	13 (81.3)	1 (reference)	1 (reference)	86 (95.6)	1 (reference)	1 (reference)
Cys/Ser	3 (18.8)	0.96 (0.21–4.48)	0.94 (0.19–4.62)	3 (3.3)	**0.26 (0.08–0.91) ^b^**	**0.25 (0.07–0.88) ^b^**
Ser/Ser	0 (0.0)	--	--	1 (1.1)	1.85 (0.11–29.93)	1.70 (0.10–27.92)
Dominant model	3 (18.8)	0.96 (0.21–4.48)	0.94 (0.19–4.62)	4 (4.4)	0.34 (0.11–1.01)	0.32 (0.11–0.97) ^b^
Recessive model	0 (0.0)	--	--	1 (1.1)	2.02 (0.13–32.71)	1.92 (0.12–31.53)
***ERCC5*** **rs17655**	16 (100)			89 (100)		
Asp/Asp	10 (62.5)	1 (reference)	1 (reference)	41 (46.1)	1 (reference)	1 (reference)
Asp/His	5 (31.3)	0.61 (0.17–2.20)	0.63 (0.17–2.34)	45 (50.6)	1.38 (0.81–2.33)	1.36 (0.80–2.30)
His/His	1 (6.3)	--	--	3 (3.4)	0.31 (0.09–1.10)	0.32 (0.09–1.14)
Dominant model	6 (37.5)	0.73 (0.21–2.51)	0.76 (0.22–2.67)	48 (53.9)	1.13 (0.68–1.88)	1.13 (0.68–1.89)
Recessive model	1 (6.3)	--	--	3 (3.4)	**0.27 (0.08–0.92) ^b^**	**0.27 (0.08–0.95) ^b^**
***XPC*** **rs2228001**	16 (100)			90 (100)		
Lys/Lys	9 (56.3)	1 (reference)	1 (reference)	30 (33.3)	1 (reference)	1 (reference)
Lys/Gln	6 (37.5)	0.58 (0.17–2.05)	0.59 (0.16–2.20)	41 (45.6)	1.01 (0.57–1.78)	1.05 (0.59–1.86)
Gln/Gln	1 (6.3)	1.56 (0.09–28.15)	1.22 (0.06–23.58)	19 (21.1)	2.05 (0.96–4.36)	2.05 (0.96–4.38)
Dominant model	7 (43.8)	0.64 (0.19–2.16)	0.63 (0.18–2.27)	60 (66.7)	1.20 (0.71–2.05)	1.24 (0.72–2.12)
Recessive model	1 (6.3)	2.00 (0.12–34.24)	1.55 (0.09–28.35)	19 (21.1)	**2.04 (1.03–4.03) ^b^**	**2.00 (1.01–3.96) ^b^**
***MSH6*** **rs1042821**	16 (100)			90 (100)		
Gly/Gly	11 (68.8)	1 (reference)	1 (reference)	57 (63.3)	1 (reference)	1 (reference)
Gly/Glu	4 (25.0)	0.86 (0.21–3.54)	0.96 (0.20–4.52)	26 (28.9)	0.70 (0.41–1.22)	0.70 (0.40–1.22)
Glu/Glu	1 (6.3)	0.86 (0.07–10.66)	1.08 (0.07–16.53)	7 (7.8)	**4.42 (1.10–17.75) ^b^**	**4.78 (1.17–19.56) ^b^**
Dominant model	5 (31.2)	0.86 (0.23–3.19)	0.98 (0.23–4.24)	33 (36.7)	0.86 (0.51–1.44)	0.86 (0.51–1.45)
Recessive model	1 (6.3)	0.90 (0.08–10.77)	1.09 (0.08–15.61)	7 (7.8)	**5.00 (1.26–19.84) ^b^**	**5.42 (1.34–21.92) ^b^**
***XRCC3* rs861539**	16 (100)			90 (100)		
Thr/Thr	8 (50.0)	1 (reference)	1 (reference)	28 (31.1)	1 (reference)	1 (reference)
Thr/Met	6 (37.5)	0.69 (0.19–2.59)	0.62 (0.16–2.43)	38 (42.2)	0.80 (0.44–1.43)	0.81 (0.45–1.46)
Met/Met	2 (12.5)	0.60 (0.09–3.89)	0.47 (0.07–3.28)	24 (26.7)	**2.26 (1.09–4.71) ^b^**	**2.36 (1.12–4.97) ^b^**
Dominant model	8 (50.0)	0.67 (0.20–2.26)	0.58 (0.16–2.08)	62 (68.9)	1.06 (0.62–1.83)	1.08 (0.63–1.88)
Recessive model	2 (12.5)	0.71 (0.12–4.18)	0.60 (0.10–3.67)	24 (26.7)	**2.60 (1.36–4.95) ^b^**	**2.68 (1.39–5.18) ^b^**
						
**Genotype**	**<50 years**			**≥50 years**		
	***n*** **(%)**	**OR (95% CI)**	**Adjusted OR (95% CI) ^a^**	***n*** **(%)**	**OR (95% CI)**	**Adjusted OR (95% CI) ^a^**
***CCNH* rs2230641**	42 (100)			64 (100)		
Val/Val	27 (64.3)	1 (reference)	1 (reference)	33 (51.6)	1 (reference)	1 (reference)
Val/Ala	14 (33.3)	0.96 (0.43–2.13)	0.93 (0.41–2.12)	29 (45.3)	**2.97 (1.55–5.68) ^b^**	**2.91 (1.51–5.60) ^b^**
Ala/Ala	1 (2.4)	0.27 (0.03–2.26)	0.27 (0.03–2.31)	2 (3.1)	5.94 (0.52–67.64)	8.01 (0.62–102.77)
Dominant model	15 (35.7)	0.82 (0.38–1.76)	0.79 (0.36–1.75)	31 (48.4)	**3.07 (1.62–5.81) ^b^**	**3.04 (1.59–5.81) ^b^**
Recessive model	1 (2.4)	0.27 (0.03–2.26)	0.27 (0.03–2.33)	2 (3.1)	4.10 (0.36–46.05)	5.67 (0.45–72.01)
***ERCC6*** **rs2228529**	42 (100)			62 (100)		
Gln/Gln	20 (47.6)	1 (reference)	1 (reference)	46 (74.2)	1 (reference)	1 (reference)
Gln/Arg	20 (47.6)	1.19 (0.56–2.54)	1.09 (0.50–2.36)	15 (24.2)	**0.49 (0.25–0.98) ^b^**	**0.48 (0.24–0.97) ^b^**
Arg/Arg	2 (4.8)	2.20 (0.29–16.75)	2.12 (0.27–16.60)	1 (1.6)	0.32 (0.04–2.84)	0.30 (0.03–2.63)
Dominant model	22 (52.4)	1.24 (0.59–2.61)	1.14 (0.53–2.44)	16 (25.8)	**0.48 (0.24–0.93) ^b^**	**0.47 (0.24–0.92) ^b^**
Recessive model	2 (4.8)	2.03 (0.28–14.91)	2.04 (0.27–15.33)	1 (1.6)	0.40 (0.05–3.53)	0.37 (0.04–3.28)
***XPC*** **rs2228001**	42 (100)			64 (100)		
Lys/Lys	17 (40.5)	1 (reference)	1 (reference)	22 (34.4)	1 (reference)	1 (reference)
Lys/Gln	15 (35.7)	0.58 (0.25–1.32)	0.58 (0.25–1.37)	32 (50.0)	1.22 (0.63–2.35)	1.27 (0.66–2.48)
Gln/Gln	10 (23.8)	2.21 (0.73–6.65)	2.11 (0.68–6.58)	10 (15.6)	1.69 (0.65–4.38)	1.74 (0.66–4.57)
Dominant model	25 (59.5)	0.82 (0.38–1.75)	0.81 (0.37–1.78)	42 (65.6)	1.31 (0.70–2.44)	1.36 (0.72–2.56)
Recessive model	10 (23.8)	**2.97 (1.07–8.21) ^b^**	**2.86 (1.01–8.08) ^b^**	10 (15.6)	1.51 (0.63–3.61)	1.52 (0.63–3.67)
***RAD51*** **rs1801321**	42 (100)			64 (100)		
G/G	14 (33.3)	1 (reference)	1 (reference)	14 (21.9)	1 (reference)	1 (reference)
G/T	19 (45.2)	0.95 (0.41–2.24)	1.00 (0.42–2.38)	31 (48.4)	1.76 (0.84–3.69)	1.83 (0.87–3.86)
T/T	9 (21.4)	0.80 (0.29–2.20)	0.75 (0.27–2.10)	19 (29.7)	**2.90 (1.23–6.83) ^b^**	**2.99 (1.25–7.14) ^b^**
Dominant model	28 (66.7)	0.90 (0.41–1.98)	0.91 (0.41–2.02)	50 (78.1)	**2.07 (1.04–4.14) ^b^**	**2.14 (1.06–4.32) ^b^**
Recessive model	9 (21.4)	0.82 (0.34–1.99)	0.75 ( (0.30–1.84)	19 (29.7)	**2.03 (1.00–4.12) ^b^**	2.05 (1.00–4.21)
***XRCC3* rs861539**	42 (100)			64 (100)		
Thr/Thr	15 (35.7)	1 (reference)	1 (reference)	21 (32.8)	1 (reference)	1 (reference)
Thr/Met	16 (38.1)	0.65 (0.27–1.52)	0.63 (0.27–1.52)	28 (43.8)	0.85 (0.43–1.68)	0.87 (0.44–1.73)
Met/Met	11 (26.2)	1.47 (0.53–4.08)	1.48 (0.52–4.19)	15 (23.4)	2.25 (0.92–5.49)	2.42 (0.97–6.03)
Dominant model	27 (64.3)	0.84 (0.38–1.83)	0.83 (0.37–1.84)	43 (67.2)	1.09 (0.57–2.05)	1.12 (0.59–2.14)
Recessive model	11 (26.2)	1.88 (0.76–4.67)	1.92 (0.77–4.83)	15 (23.4)	**2.47 (1.11–5.51) ^b^**	**2.63 (1.16–5.97) ^b^**
***XRCC5*** **rs2440**	42 (100)			62 (100)		
C/C	8 (19.0)	1 (reference)	1 (reference)	22 (35.5)	1 (reference)	1 (reference)
C/T	23 (54.8)	2.25 (0.88–5.77)	2.53 (0.96–6.62)	31 (50.0)	1.00 (0.52–1.95)	0.97 (0.50–1.90)
T/T	11 (26.2)	2.35 (0.79–6.98)	2.53 (0.84–7.63)	9 (14.5)	1.28 (0.49–3.38)	1.29 (0.48–3.45)
Dominant model	34 (81.0)	2.28 (0.94–5.57)	**2.53 (1.02–6.26) ^b^**	40 (64.5)	1.06 (0.56–1.99)	1.03 (0.54–1.95)
Recessive model	11 (26.2)	1.38 (0.58–3.29)	1.41 (0.58–3.43)	9 (14.5)	1.28 (0.53–3.11)	1.31 (0.53–3.23)

^a^ ORs were adjusted for gender (male and female), age (<30, 30–49, 50–69, and ≥70 years), and smoking status (non-smoker and smoker). ^b^ Significant results (p < 0.05) highlighted in bold. Abbreviations: DTC, well–differentiated thyroid cancer; SNP, single nucleotide polymorphism; OR, odds ratio; CI, confidence interval.

**Table 5:** Risk score (RS) in case and control populations and associated DTC risk (crude and adjusted ORs). Risk scores calculated from significant results on single SNP analysis^a^.

Risk Score (RS) ^a.^	Frequency		*p*-Value ^b^	OR (95% CI)	*p*-Value	Adjusted OR (95% CI) ^c^	*p*-Value ^b^
	Controls *n* (%)	Cases *n* (%)					
**DTC (all cases)**	**191 (100)**	**106 (100)**					
0–1	114 (59.7)	34 (32.1)	**<0.001 ^d^**	1 (Reference)		1 (Reference)	
2	64 (33.5)	52 (49.1)		**2.72 (1.60** **–** **4.63) ^d^**	**<0.001 ^d^**	**2.68 (1.56–4.59) ^d^**	**<0.001 ^d^**
3/+	13 (6.8)	20 (18.9)		**5.16 (2.33–11.44) ^d^**	**<0.001 ^d^**	**5.02 (2.24–11.24) ^d^**	**<0.001 ^d^**
**Histological type**							
**Papillary TC**	152 (100)	78 (100)					
0–2	85 (55.9)	17 (21.8)	**<0.001 ^d^**	1 (Reference)		1 (Reference)	
3	48 (31.6)	44 (56.4)		**4.58 (2.36–8.89) ^d^**	**<0.001 ^d^**	**4.55 (2.34–8.84) ^d^**	**<0.001 ^d^**
4/+	19 (12.5)	17 (21.8)		**4.47 (1.94–10.32) ^d^**	**<0.001 ^d^**	**4.46 (1.92–10.36) ^d^**	**<0.001 ^d^**
**Follicular TC**	56 (100)	28 (100)					
0–1	24 (42.9)	5 (17.9)	**0.029 ^d^**	1 (Reference)		1 (Reference)	
2/+	32 (57.1)	23 (82.1)		**3.45 (1.15–10.39) ^d^**	**0.028 ^d^**	**3.52 (1.12–11.07) ^d^**	**0.032 ^d^**
**Gender**							
**Female**	174 (100)	89 (100)					
0–2	114 (65.5)	28 (31.5)	**<0.001 ^d^**	1 (Reference)		1 (Reference)	
3	51 (29.3)	43 (48.3)		**3.43 (1.92–6.13) ^d^**	**<0.001 ^d^**	**3.42 (1.90–6.14) ^d^**	**<0.001 ^d^**
4/+	9 (5.2)	18 (20.2)		**8.14 (3.31–20.04) ^d^**	**<0.001 ^d^**	**8.01 (3.22–19.92) ^d^**	**<0.001 ^d^**
**Age**							
**<50 years**	83 (100)	42 (100)					
0	26 (31.3)	6 (14.3)	**0.020 ^d^**	1 (Reference)		1 (Reference)	
1	52 (62.7)	28 (66.7)		2.33 (0.86–6.34)	0.097	2.52 (0.92–6.94)	0.073
2	5 (6.0)	8 (19.0)		**6.93 (1.66–28.89) ^d^**	**0.008 ^d^**	**7.34 (1.72–31.24) ^d^**	**0.007 ^d^**
**≥50 years**	127 (100)	62 (100)					
0–1	60 (47.2)	12 (19.4)	**<0.001 ^d^**	1 (Reference)		1 (Reference)	
2	51 (40.2)	26 (41.9)		**2.55 (1.17–5.56) ^d^**	**0.019 ^d^**	**2.66 (1.21–5.85) ^d^**	**0.015 ^d^**
3/+	16 (12.6)	24 (38.7)		**7.50 (3.09–18.18) ^d^**	**<0.001 ^d^**	**7.90 (3.21–19.45) ^d^**	**<0.001 ^d^**

^a^ For the purpose of risk score calculations, genotypes presenting significant results on single SNP analysis were attributed a +1 score, risk score for each participant corresponding to the sum of such scores (+1 in all cases: *CCNH* rs2230641 Val/Ala or Ala/Ala + *ERCC5* rs2227869 Cys/Cys or Ser/Ser + *XPC* rs2228001 Gln/Gln + *MSH6* rs1042821 Glu/Glu + *XRCC3* rs861539 Met/Met; +1 in papillary TC: *MUTYH* rs3219489 Gln/Gln + *ERCC5* rs2227869 Cys/Cys + *XPC* rs2228001 Gln/Gln + *NBN* rs1805794 Glu/Glu or Glu/Gln + *XRCC3* rs861539 Met/Met; +1 in follicular TC: *MLH3* rs175080 Pro/Leu or Leu/Leu + *MSH6* rs1042821 Glu/Glu + *XRCC2* rs3218536 Arg/Arg; +1 in female participants: *CCNH* rs2230641 Val/Ala or Ala/Ala + *ERCC5* rs2227869 Cys/Cys + *ERCC5* rs17655 Asp/Asp or Asp/His + *XPC* rs2228001 Gln/Gln + *MSH6* rs1042821 Glu/Glu + *XRCC3* rs861539 Met/Met; +1 in participants with age <50 years: *XPC* rs2228001 Gln/Gln + *XRCC5* rs2440 C/T or T/T; +1 in participants with age ≥50 years: *CCNH* rs2230641 Val/Ala or Ala/Ala + *ERCC6* rs2228529 Gln/Gln + *RAD51* rs1801321 G/T or T/T + *XRCC3* rs861539 Met/Met). ^b^ *p*–value for cases *versus* control group determined by two–sided Fisher’s exact test (whenever 2 × 2 contingency tables are possible) or χ^2^ test (remaining cases). ^ c^ ORs were adjusted for gender (male and female), age (<30, 30–49, 50–69, ≥70 years) and smoking status (non–smoker and smoker). ^d^ *p* < 0.05. Abbreviations: DTC, well–differentiated thyroid cancer; MAF, minor allele frequency; OR, odds ratio; CI, confidence interval.

**Table 6:** Two-way SNP interactions among DNA repair genes: distribution of combined genotypes in enrolled populations and associated DTC risk (adjusted ORs). Only SNPs presenting significant findings (*p* < 0.01) are shown.

Combined Genotype	Frequency			DTC Risk	
	Controls *n* (%)	Cases *n* (%)	*p*-Value ^a^	Adjusted OR (95% CI) ^b^	*p*-Value ^a^
***CCNH* rs2230641 – *RAD51* rs1801321**	212 (100)	106 (100)			
Val/Val – G/G	58 (27.4)	13 (12.3)	0.037 ^c^	1 (Reference)	
Val/Val – G/T	64 (30.2)	29 (27.4)		2.10 (0.99–4.45)	0.052
Val/Ala – G/G	15 (7.1)	13 (12.3)		**3.77 (1.44–9.87)**	**0.007 ^d^**
Val/Ala – G/T	27 (12.7)	20 (18.9)		**3.43 (1.46–8.06)**	**0.005 ^d^**
Val/Val – T/T	26 (12.3)	18 (17.0)		3.05 (1.29–7.19)	0.011 ^c^
Val/Ala – T/T	14 (6.6)	10 (9.4)		3.22 (1.17–8.89)	0.024 ^c^
Ala/Ala – G/G Ala/Ala – G/T Ala/Ala – T/T	8 (3.8)	3 (2.8)		1.86 (0.42–8.18)	0.414
***MUTYH* rs3219489 –** ***CCNH* rs2230641**	211 (100)	106 (100)			
Gln/Gln – Val/Val	77 (36.5)	35 (33.0)	0.018 ^c^	1 (Reference)	
Gln/Gln – Val/Ala	22 (10.4)	26 (24.5)		**2.68 (1.32–5.42)**	**0.006 ^d^**
Gln/His – Val/Val	66 (31.3)	23 (21.7)		0.81 (0.43–1.51)	0.500
Gln/His – Val/Ala	30 (14.2)	14 (13.2)		1.05 (0.49–2.23)	0.904
Gln/Gln – Ala/Ala His/His – Val/Val Gln/His – Ala/Ala His/His – Val/Ala	16 (7.6)	8 (7.5)		1.24 (0.48–3.23)	0.660
***CCNH* rs2230641 – *MLH3* rs175080**	195 (100)	106 (100)			
Val/Val – Pro/Pro	40 (20.5)	11 (10.4)	0.097	1 (Reference)	
Val/Val – Pro/Leu	77 (39.5)	36 (34.0)		1.76 (0.80–3.87)	0.162
Val/Ala – Pro/Pro	14 (7.2)	11 (10.4)		2.60 (0.91–7.41)	0.074
Val/Ala – Pro/Leu	23 (11.8)	21 (19.8)		**3.34 (1.35–8.26)**	**0.009 ^d^**
Val/Val – Leu/Leu	25 (12.8)	13 (12.3)		1.95 (0.75–5.09)	0.173
Val/Ala – Leu/Leu	11 (5.6)	11 (10.4)		3.69 (1.25–10.90)	0.018 ^c^
Ala/Ala – Pro/Pro Ala/Ala – Pro/Leu Ala/Ala – Leu/Leu	5 (2.6)	3 (2.8)		2.44 (0.48–12.45)	0.284
***CCNH* rs2230641 – *MSH4* rs5745549**	195 (100)	106 (100)			
Val/Val – Ser/Ser	132 (67.7)	51 (48.1)	**0.009 ^d^**	1 (Reference)	
Val/Val – Ser/Asn	10 (5.1)	9 (8.5)		2.45 (0.93–6.43)	0.070
Val/Ala – Ser/Ser	41 (21.0)	38 (35.8)		**2.27 (1.30–3.96)**	**0.004 ^d^**
Val/Ala – Ser/Asn Ala/Ala – Ser/Ser	12 (6.2)	8 (7.5)		1.87 (0.71–4.92)	0.207
***MLH3* rs175080 – *RAD51* rs1801321**	195 (100)	106 (100)			
Pro/Pro – G/G	23 (11.8)	4 (3.8)	0.288	1 (Reference)	
Pro/Pro – G/T	24 (12.3)	10 (9.4)		2.88 (0.77–10.78)	0.117
Pro/Leu – G/G	32 (16.4)	18 (17.0)		3.98 (1.14–13.89)	0.031 ^c^
Pro/Leu – G/T	46 (23.6)	25 (23.6)		3.59 (1.09–11.81)	0.035 ^c^
Pro/Pro – T/T	9 (4.6)	8 (7.5)		5.43 (1.23–23.88)	0.025 ^c^
Leu/Leu – G/G	14 (7.2)	6 (5.7)		2.92 (0.68–12.57)	0.151
Pro/Leu – T/T	23 (11.8)	16 (15.1)		4.66 (1.32–16.45)	0.017 ^c^
Leu/Leu – G/T	16 (8.2)	15 (14.2)		**6.22 (1.70–22.78)**	**0.006 ^d^**
Leu/Leu – T/T	8 (4.1)	4 (3.8)		3.55 (0.69–18.15)	0.128
***ERCC6*** **rs4253211 –** ***RAD51* rs1801321**	211 (100)	102 (100)			
Arg/Arg – G/G	65 (30.8)	16 (15.7)	0.026 ^c^	1 (Reference)	
Arg/Arg – G/T	72 (34.1)	42 (41.2)		**2.51 (1.28–4.94)**	**0.007 ^d^**
Arg/Pro – G/T	21 (10.0)	7 (6.9)		1.53 (0.54–4.29)	0.423
Arg/Arg – T/T	33 (15.6)	21 (20.6)		2.67 (1.22–5.85)	0.014 ^c^
Arg/Pro – G/G Pro/Pro – G/G Arg/Pro – T/T Pro/Pro – G/T	20 (9.5)	16 (15.7)		**3.65 (1.52–8.78)**	**0.004 ^d^**
***MLH3* rs175080 – *MSH6* rs1042821**	210 (100)	106 (100)			
Pro/Pro – Gly/Gly	32 (15.2)	19 (17.9)	0.032 ^c^	1 (Reference)	
Pro/Pro – Gly/Glu	26 (12.4)	2 (1.9)		**0.11 (0.02–0.53)**	**0.006 ^d^**
Pro/Leu – Gly/Gly	71 (33.8)	36 (34.0)		0.81 (0.40–1.65)	0.561
Pro/Leu – Gly/Glu	35 (16.7)	19 (17.9)		0.94 (0.41–2.13)	0.878
Leu/Leu – Gly/Gly	24 (11.4)	13 (12.3)		0.83 (0.34–2.03)	0.680
Leu/Leu – Gly/Glu	17 (8.1)	9 (8.5)		0.89 (0.33–2.43)	0.819
Pro/Pro – Glu/Glu Pro/Leu – Glu/Glu Leu/Leu – Glu/Glu	5 (2.4)	8 (7.5)		3.09 (0.85–11.27)	0.088
***MSH4* rs5745549 – *MSH6* rs1042821**	210 (100)	106 (100)			
Ser/Ser – Gly/Gly	124 (59.0)	60 (56.6)	**0.004 ^d^**	1 (Reference)	
Ser/Ser – Gly/Glu	63 (30.0)	24 (22.6)		0.81 (0.46–1.43)	0.467
Ser/Asn – Gly/Glu	15 (7.1)	6 (5.7)		0.83 (0.30–2.28)	0.720
Ser/Asn – Gly/Gly Ser/Ser – Glu/Glu	8 (3.8)	16 (15.1)		**4.63 (1.83–11.69)**	**0.001 ^d^**
***ERCC6*** **rs4253211 –** ***MLH3* rs175080**	195 (100)	102 (100)			
Arg/Arg – Pro/Pro	51 (26.2)	13 (12.7)	0.067	1 (Reference)	
Arg/Arg – Pro/Leu	78 (40.0)	45 (44.1)		2.43 (1.18–5.04)	0.017 ^c^
Arg/Pro – Pro/Leu	21 (10.8)	10 (9.8)		2.25 (0.83–6.14)	0.113
Arg/Arg – Leu/Leu	30 (15.4)	21 (20.6)		2.96 (1.28–6.88)	0.012 ^c^
Arg/Pro – Pro/Pro Pro/Pro – Pro/Pro Arg/Pro – Leu/Leu Pro/Pro – Pro/Leu Pro/Pro – Leu/Leu	15 (7.7)	13 (12.7)		**4.23 (1.55–11.53)**	**0.005 ^d^**
***RAD51* rs1801321 – *XRCC3* rs861539**	209 (100)	106 (100)			
G/G – Thr/Thr	26 (12.4)	7 (6.6)	**0.006 ^d^**	1 (Reference)	
G/G – Thr/Met	35 (16.7)	15 (14.2)		1.59 (0.56–4.49)	0.381
G/T – Thr/Thr	29 (13.9)	24 (22.6)		3.10 (1.14–8.44)	0.027 ^c^
G/T – Thr/Met	55 (26.3)	14 (13.2)		0.98 (0.35–2.73)	0.967
G/G – Met/Met	11 (5.3)	6 (5.7)		1.99 (0.54–7.41)	0.304
T/T – Thr/Thr	15 (7.2)	5 (4.7)		1.23 (0.33–4.61)	0.759
G/T – Met/Met	12 (5.7)	12 (11.3)		3.77 (1.17–12.13)	0.026 ^c^
T/T – Thr/Met	22 (10.5)	15 (14.2)		2.41 (0.83–7.05)	0.108
T/T – Met/Met	4 (1.9)	8 (7.5)		**7.90 (1.80–34.74)**	**0.006 ^d^**
***ERCC6*** **rs2228529 –** ***MSH4* rs5745549**	195 (100)	104 (100)			
Gln/Gln – Ser/Ser	102 (52.3)	53 (51.0)	**0.009 ^d^**	1 (Reference)	
Gln/Gln – Ser/Asn	6 (3.1)	13 (12.5)		**4.77 (1.67–13.61)**	**0.003 ^d^**
Gln/Arg – Ser/Ser	71 (36.4)	34 (32.7)		0.82 (0.48–1.43)	0.489
Gln/Arg – Ser/Asn Arg/Arg – Ser/Ser Arg/Arg – Ser/Asn	16 (8.2)	4 (3.8)		0.46 (0.14–1.47)	0.190
***MSH4* rs5745549 – *XRCC5* rs2440**	195 (100)	104 (100)			
Ser/Ser – C/C	67 (34.4)	24 (23.1)	0.049 ^c^	1 (Reference)	
Ser/Ser – C/T	84 (43.1)	50 (48.1)		1.76 (0.97–3.19)	0.063
Ser/Asn – C/T	12 (6.2)	4 (3.8)		1.02 (0.29–3.56)	0.972
Ser/Ser – T/T	27 (13.8)	17 (16.3)		1.86 (0.84–4.12)	0.124
Ser/Asn – C/C Ser/Asn – T/T	5 (2.6)	9 (8.7)		**6.18 (1.83–20.86)**	**0.003 ^d^**
***MUTYH* rs3219489 – *XPC* rs2228001**	211 (100)	106 (100)			
Gln/Gln – Lys/Lys	38 (18.0)	28 (26.4)	0.037 ^c^	1 (Reference)	
Gln/Gln – Lys/Gln	54 (25.6)	27 (25.5)		0.68 (0.35–1.35)	0.274
Gln/His – Lys/Lys	41 (19.4)	9 (8.5)		**0.31 (0.13–0.73)**	**0.008** **^d^**
Gln/His – Lys/Gln	48 (22.7)	18 (17.0)		0.55 (0.26–1.16)	0.117
Gln/Gln – Gln/Gln	13 (6.2)	8 (7.5)		0.81 (0.29–2.25)	0.689
Gln/His – Gln/Gln	9 (4.3)	11 (10.4)		1.70 (0.61–4.77)	0.311
His/His – Lys/Lys His/His – Lys/Gln His/His – Gln/Gln	8 (3.8)	5 (4.7)		0.91 (0.26–3.16)	0.884
***MSH3*** **rs184967 – *XRCC5* rs1051685**	195 (100)	106 (100)			
Arg/Arg – A/A	99 (50.8)	70 (66.0)	**0.001 ^d^**	1 (Reference)	
Arg/Arg – A/G	32 (16.4)	8 (7.5)		0.34 (0.15–0.80)	0.013 ^c^
Arg/Gln – A/A	52 (26.7)	14 (13.2)		**0.36 (0.18–0.71)**	**0.003 ^d^**
Arg/Gln – A/G Arg/Arg – G/G Gln/Gln – A/A	12 (6.2)	14 (13.2)		1.46 (0.62–3.40)	0.387
***CCNH* rs2230641 – *LIG4* rs1805388**	212 (100)	106 (100)			
Val/Val – Thr/Thr	112 (52.8)	42 (39.6)	0.015 ^c^	1 (Reference)	
Val/Val – Thr/Ile	32 (15.1)	16 (15.1)		1.36 (0.67–2.75)	0.396
Val/Ala – Thr/Thr	37 (17.5)	36 (34.0)		**2.62 (1.45–4.71)**	**0.001 ^d^**
Val/Ala – Thr/Ile	18 (8.5)	5 (4.7)		0.73 (0.25–2.11)	0.555
Val/Val – Ile/Ile Ala/Ala – Thr/Thr Val/Ala – Ile/Ile Ala/Ala – Thr/Ile	13 (6.1)	7 (6.6)		1.47 (0.53–4.08)	0.456

^a^*p* value for cases *versus* control group determined by two–sided Fisher’s exact test (whenever 2x2 contingency tables are possible) or χ^2^ test (remaining cases). ^ b^ ORs were adjusted for gender (male and female), age (<30, 30–49, 50–69, ≥ 70 years) and smoking status (non-smoker and smoker). ^c^ p<0.05. ^d^*p* < 0.01.

**Table 7:** Haplotypes comprising SNPs located in the same chromosome arm and corresponding DTC risk (adjusted ORs). Only haplotypes presenting significant results are shown.

Haplotype						Adj. OR (95% CI)	*p*-Value ^a^
Chromosome 5q							
*CCNH* rs2230641	*CDK7* rs2972388	*MSH3* rs26279	*MSH3* rs184967	*XRCC4* rs1805377	*XRCC4* rs28360135		0.015
Val	A	Thr	Arg	G	Ile	1.00 (Reference)	
Val	A	Ala	Arg	G	Ile	0.26 (0.08–0.87)	**0.03**
Val	G	Ala	Gln	G	Ile	0.15 (0.03–0.72)	**0.019**

^a^*p* < 0.05. Abbreviations: DTC, well-differentiated thyroid cancer; OR, odds ratio; CI, confidence interval.
